# MicroRNAs as Modulators of Oral Tumorigenesis—A Focused Review

**DOI:** 10.3390/ijms22052561

**Published:** 2021-03-04

**Authors:** Kumar Rishabh, Soham Khadilkar, Aviral Kumar, Ishu Kalra, Alan Prem Kumar, Ajaikumar B. Kunnumakkara

**Affiliations:** 1 Cancer Biology Laboratory and DBT-AIST International Center for Translational and Environmental Research (DAICENTER), Department of Biosciences and Bioengineering, Indian Institute of Technology (IIT) Guwahati, Guwahati, Assam 781039, India; rishabh18b@iitg.ac.in (K.R.); khadilka@iitg.ac.in (S.K.); aviral.kumar@iitg.ac.in (A.K.); ishukalraish2@gmail.com (I.K.); 2 Cancer Science Institute of Singapore, Department of Pharmacology, Yong Loo Lin School of Medicine, National University of Singapore, Singapore 117599, Singapore; 3 NUS Centre for Cancer Research (N2CR), Yong Loo Lin School of Medicine, National University of Singapore, Singapore 117593, Singapore; 4 National University Cancer Institute, National University Health System, Singapore 119074, Singapore

**Keywords:** oral cancers, miRNAs, non-coding RNAs, invasion, apoptosis, metastasis

## Abstract

Oral cancers constitute the majority of head and neck tumors, with a relatively high incidence and poor survival rate in developing countries. While the five-year survival rates of the oral cancer patients have increased to 65%, the overall survival for advanced stages has been at 27% for the past ten years, emphasizing the necessity for further understanding the etiology of the disease, diagnosis, and formulating possible novel treatment regimens. MicroRNAs (miRNAs), a family of small non-coding RNA, have emerged as master modulators of gene expression in various cellular and biological process. Aberrant expression of these dynamic molecules has been associated with many human diseases, including oral cancers. The deregulated miRNAs have been shown to control various oncogenic processes, including sustaining proliferative signaling, evading growth suppressors, resisting cell death activating invasion and metastasis, and inducing angiogenesis. Hence, the aberrant expression of miRNAs associated with oral cancers, makes them potential candidates for the investigation of functional markers, which will aid in the differential diagnosis, prognosis, and development of novel therapeutic regimens. This review presents a holistic insight into our understanding of the role of miRNAs in regulating various hallmarks of oral tumorigenesis.

## 1. Introduction 

Oral cancer constitutes a part of the head and neck squamous carcinoma (HNSCC), and is one of the most common cancers afflicting millions worldwide [1,2]. It mostly arises from the mucosal epithelial lining of the oral cavity, which includes the anterior of the tongue, buccal cavity, lips, and lower alveolar ridges [3,4]. Although the five-year survival rate is increased to 65%, diagnosis at advanced stages leads to a five-year survival of only 27%, necessitating the need for the development of early diagnosis and novel therapeutic approaches in combating this disease [5,6,7]. Tobacco chewing and alcohol consumption are the major risk factors associated with oral cancers [8]. Other factors include the chewing of areca nuts, poor oral health hygiene, betel leaf, and human papillomavirus (HPV) infection [9,10]. With the change in lifestyle and environmental factors, the incidence of oral cancer is increasing at an unprecedented rate in developing countries [11,12]. Treatment modalities mostly rely on the position of tumor, identification of risk type based on histopathology, and other comorbidities. At present, conventional therapeutic approaches for oral cancers involve surgical resection at tumor sites, with or without neck dissection or chemotherapy, followed by adjuvant radiotherapy [13]. These approaches present poor clinical outcomes in late stages with recurrence and distal metastasis being the most common [12,14,15,16]. Thus, a molecular-level understanding of the tumor dynamics, its heterogenicity, and its pathophysiology are imperative in the development of novel and more efficacious therapies.

The discovery of small non-coding RNA molecules in gene regulation has revolutionized the clinical realm, providing newer impetus in the identification of their mechanistic role in biological processes and human disease conditions. miRNAs are evolutionarily conserved, endogenous, small non-coding RNA molecules of about 18–24-nt in length that function as post-transcriptional gene regulators [17]. Since its identification in 1993 as small RNA molecule in *Caenorhabditis elegans*, it has been extensively studied and well-characterized over the past decades [18]. miRNAs are known to regulate various biological processes, such as proliferation, differentiation, apoptosis, immune response, and maintenance of homeostasis [19,20,21,22,23]. These small dynamic RNA molecules regulate gene expression by binding to the 3′-untranslated regions (3′-UTR) of target mRNAs, leading to post-transcriptional inactivation of the target gene, either by mRNA degradation or inhibiting translation [24]. Due to its variability in the binding region and imperfect complementary binding, a single miRNA can bind and target multiple mRNAs [25]. Dysregulation of these non-coding RNAs, due to genetic and epigenetic events, is found to disrupt the balance of these pleiotropically acting molecules, in turn contributing to many human disorders, including cancers [26,27]. Several studies have shown significant insight into miRNA’s role in tumor development and progression since its first discovery in cancer [28]. A regulator of cell proliferation, apoptosis, invasion, migration, and stem cell maintenance, this noncoding RNA molecule, through recent studies, has also emerged as a controller of tumor microenvironment (TME) remodeling and tumor metastasis [29]. Primarily, two classes of miRNAs, oncomiRs and tumor suppressor miRs (TS-miRs), are observed that regulate the expression of oncogenes and tumor suppressor genes, respectively [30]. Accumulating evidence has shown insight into the functional role of the deregulated miRNAs in controlling the expression of their target mRNAs, leading to regulation of signaling pathways involved in oral cancer [31,32,33]. A total of 2300 true human mature miRNAs have been identified, but their action on target mRNAs is challenging to characterize due to their multi-target inherent nature. Although bioinformatics tools using different algorithms and parameters are used to analyze effective miRNA: mRNA interaction, their prediction accuracy is questionable [34]. Furthermore, miRNAs have been reported to possess unique signatures in different cancer types, grades and stages of tumors, making them a promising diagnostic and prognostic marker to detect the change in normal cellular function within the tumor tissues [35,36]. Hence, a deeper understanding of these dynamic molecules holds the key to the mechanism of oral cancer initiation and progression, which could further help in the clinical management of oral cancer. Through this review, we will provide insight into the role of these pleiotropically acting RNA molecules at different levels of hallmarks in oral tumorigenesis and reflect on the chances of understanding and manipulating these candidate molecules to develop newer diagnostic and therapeutic strategies.

## 2. MicroRNA Biogenesis

MiRNAs are highly conserved, small non-coding RNAs responsible for the modulation of 60% of protein-coding gene expression in the human genome, at the translational level. Increasing experimental evidence report miRNA genes to be distributed all along the genome [37,38]. In humans, about half of the identified miRNAs are found as clusters that are transcribed as polycistronic primary transcripts. miRNAs are present in four different regions namely, genomic clusters, i.e., exons of protein-coding regions, introns of protein-coding regions, and introns and exons of non-coding transcription units, but few of them are also found in long interspersed nuclear elements, such as repetitive sequences [39].

**Figure 1:**
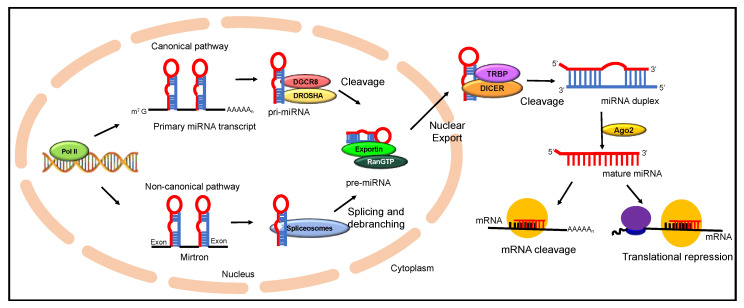
A schematic illustrating the miRNA biogenesis and its regulation of gene expression by post-transcriptional inactivation.

miRNA biosynthesis is an extremely well-coordinated multi-step sequential process that begins in the nucleus and ends in the cytoplasm, where miRNA carries out its primary function of post-transcriptional inactivation (Figure 1) [40]. They are transcribed by RNA polymerase II leading to the production of pri-miRNAs, which are precursor RNAs of several kilobases in length [41]. More than 30% of pri-miRNAs are transcribed from introns of protein-coding genes, while some miRNAs are produced from specialized miRNA gene loci. An individual pri-miRNA can either form a single miRNA or produce a different cluster of miRNAs formed from a common miRNA primary transcript [42]. Drosha, a microprocessor enzyme of the RNase III enzyme family along with DiGeorge syndrome chromosomal (or critical) region 8 (DGCR8), a double-stranded RNA binding protein, is recruited for further processing of the capped and polyadenylated pri-miRNA. Processing of pri-mRNA results in the formation of pre-miRNAs, which are approximately 70 nucleotide segments folded into stem-loop structures [43]. This is followed by a guanosine triphosphate (GTP)-dependent transport of pre-miRNA by exportin-5 to the cytoplasm. In the cytoplasm, DICER1, and RNase III enzyme, further processes the pre-miRNA to produce mature double-stranded RNA of nearly 22 nucleotides in length [44]. One strand of the mature miRNA is hooked to miRNA induced silencing complex (miRISC complex) and with the help of DICER1 and Argonaute (AGO), which directs it to target the complementary strand of mRNA. This is generally termed as the canonical processing of miRNA biosynthesis [45]. Nonetheless, there have been findings according to which another subset of miRNA, termed as mirtrons, which are pre-miRNA-like hairpin structures, undergo splicing and debranching by evading the Drosha processing step. These structures directly enter the pathway at as exportin-5 substrates. This mechanism is commonly known as the non-canonical pathway of miRNA processing which is observed in miRNA production using various other microprocessor molecules [46]. Though most miRNAs act in a RISC dependent manner, a confined number of miRNAs in the nucleus, are known to work non-canonically by demonstrating RISC independent activity. One major form of non-canonical pathway for miRNA biosynthesis is the Mirtron pathway, where the mature miRNA is processed with the Drosha independent mechanism [47]. Here, the 5′ and 3′ ends of pre-miRNA are cleaved by spliceosomes and debranching enzymes to generate short hairpin structures. These pre-miRNA structures are then exported to cytoplasm by Exportin 5, where it is cleaved by DICER enzymes. The major difference between the two different forms of miRNA biosynthesis pathways is the bypassing of microprocessor step with the splicing machinery to merge with the canonical pathway of miRNA biogenesis [47]. Recent studies have shown variability of Mirtron within the species of mammalian and invertebrate origin in terms of splice acceptor sites, GC content and hairpin loop arrangement. Still, Mirtron has emerged as crucial regulators of gene expression and further studies are needed to elucidate the exact biogenesis and mechanistic role of these small hairpin structures in disease etiology [48]. Thus, miRNA portrays tremendous versatility in the role it plays within the cell. Through different mechanisms, miRNAs in both cytoplasm and nucleus have established their role in regulating gene expression [49]. miRNA induced silencing complex (miRISC) within the nucleus is found to regulate the expression of small RNA molecules like long non-coding RNA through post-transcriptional degradation. Studies have also revealed the role of miRNAs regulating the conversion of the pri-miRNA into pre-miRNA through feedback loop mechanisms [50]. The evidence on colocalization of miR-206 and 28s-rRNA has emanated inquiry into the role of miRNA in ribosomal biogenesis [51]. Similarly, recent research indicates miRNA being a part of the different cellular processes, such as alternate splicing and transcriptional gene activation and silencing [52]. 

## 3. MicroRNAs in Oral Cancer Pathogenesis 

**Figure 2:**
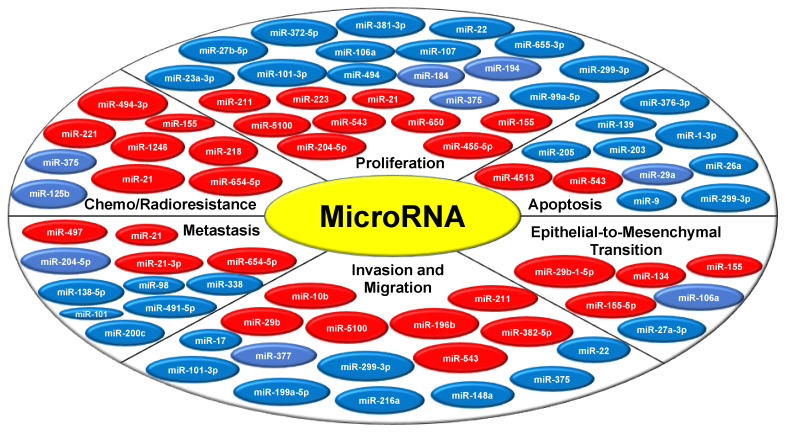
MicroRNA modulates oral tumorigenesis by regulating various hallmarks of cancer. The miRNAs represented in the red circle indicates the tumor promoting or oncogenic miRNAs and the miRNAs represented in the blue circle depicts the tumor suppressor miRNAs.

MiRNAs being the master regulator are known to modulate various hallmarks of cancer, either by acting as oncogenes or tumor suppressor genes (Figure 2). miRNA altered signatures disturb the biological balance that modulates various signaling pathways leading to disease onset and progression. A growing body of research now aims to unravel the miRNA regulatory network code involved in oral tumor pathogenesis (Table 1). From a molecular outlook, since oral cancer is complex and relates to the host pathophysiology, understanding these miRNA molecules, and their regulation would be a crucial step in developing a more targeted type of cancer therapy. 

### 3.1. Proliferation

Accumulating evidence has established the varied role of different miRNAs as essential regulators in the proliferation of oral cancer. miRNAs regulate oral cancer progression by targeting various transcription factors and proliferative pathways. Increased expression of miRNA-10a promoted oral squamous cell carcinoma (OSCC) cell proliferation through the regulation of glucose metabolism by glucose transporter 1 (GLUT1) levels. Furthermore, miRNA-10a and GLUT1 were found to be enriched in oral cancer tissues as compared to healthy controls [56]. In another study, miR-211 was found to promote proliferation in OSCC by targeting the bridging integrator 1 protein (BIN1) [127]. Mir-21 was reported to regulate the proliferation in SCC-15 cells by targeting TNF-α expression, without inducing any effect on the cellular apoptotic pathway [61]. A study identified cytochrome P450 family 3 subfamily A member 5 (CYP3A5) as a direct target of miR-543 using *in-silico* analysis and dual-luciferase reporter assays. The results of the study suggested that miR-543 serves a vital role in OSCC proliferation [155]. Another study suggested the miR-375/SLCA11 axis as a major detrimental factor in promoting proliferation in CAL-27 and Tca8113 cell lines [167]. Fang, Z. et al., (2017) demonstrated miR-184 as a tumor suppressor gene by modulating the expression of long non-coding RNA urothelial cancer-associated 1 (UCA1) and SF1. Reduction in miR-184 levels reversed the tumor-suppressive effects in OSCC cell populations [108]. 

Increasing experimental evidences have implicated that miRNAs can also act as tumor suppressor genes resulting in suppression of oral cancer progression. Knockdown of *miR-5100* suppressed proliferation of OSCC cells by increasing the populations of cells in the G1 and G2 phases, and subsequent reduction in the S phase [166]. In another study, fibroblast growth factor receptor 2 (FGFR2) was downregulated by miR-381-3p through direct interactions with its 3’ untranslated region. Knockdown of FGFR2 recapitulated the growth-suppressive effect of miR-381-3p. Conversely, restoring FGFR2 expression attenuated miR-381-3p-induced effects in OSCC cells [143]. miR-299-3p was found to inhibit oral squamous cell carcinoma cell proliferation by targeting forkhead box P4 (FOXP4) expression thereby promoting apoptosis [135]. The overexpression of miR-107 was identified to inhibit OSCC cell proliferation and target TP53 regulated inhibitor of apoptosis 1 (TRIAP1) to regulate the gene expression [85]. In another study, it was discovered that the downregulation of miR-4513 expression inhibits cell proliferation by CXCL17, which is a direct target of miR-4513. Knocking down the expression of CXCL17, inhibited the effects of miR-4513 on OSCC cell behaviors [165]. miR-494 repressed the expression of homeobox A10 (HOXA10) levels and was also observed to reduce the proliferation of oral cancer cells [168]. In a study, miR-655 was found to suppress cell proliferation in OSCC by directly targeting metadherin, a cell surface tumor-associated protein through PTEN/AKT pathway [160]. Downregulation of miR-30a-5p decreased the levels of FAP which led to the suppression of proliferation in OSCC cells [169]. An analysis of miR-101-3p showed that exosomes derived from human bone marrow mesenchymal stem cells overexpressing miR-101-3p suppressed oral cancer cell proliferation. Furthermore, it was observed that COL10A1 was upregulated, while miR-101-3p downregulated in oral tumor samples, and miR-101-3p was directly targeting COL10A1 as verified by dual-luciferase reporter gene assay [83]. Similarly, miR-133a-3p was also found to regulate COL1A1 expression levels, thereby inhibiting the proliferation in various oral cancer cell lines [89]. In another study, it was found that LIM Domain Kinase 1 (LIMK1) is a direct target of miR-106a in OSCC cells and it inhibited the cell proliferation by directly decreasing LIMK1 expression [84]. mir-27b-5p and miR-372-5p were reported to bind to the 3′UTR region of proliferating cell nuclear antigen (PCNA), hence reducing proliferation in OSCC cells [170]. Chen, F. et al. (2019) analyzed the correlation between miR-23a-3p and prognosis of oral cancer patients. It was found that fibroblast growth factor 2 (FGF2) was revealed as a direct target of miR-23a-3p, based on luciferase assays and immunoblotting. Moreover, expression of miR-23a-3p and FGF2 was found to be significantly downregulated and upregulated in OSCC tissues respectively [65]. Apart from these, other studies have implicated different miRNAs (miR-22 [64], miR-34a [77], miR-99a-5p [81], miR-138 [91,92], miR-145 [97], miR-155 [103], miR-155-5p [105], miR-188 [111], miR-194 [112], miR-195-5p [114], miR-204-5p [123], miR-211 [127], miR-216a [129], miR-223 [134], miR-340 [138], miR-455-5p [148], miR-495 [153], miR-650 [158]) to be involved in proliferation of oral cancer. Hence a plethora of literature indicated the immense role of miRNAs in regulating proliferation in oral cancers and targeting these non-coding RNA molecules can help in circumventing this deadly oral disease. 

### 3.2. Apoptosis 

Apoptosis is a naturally acquired programmed cell death, which is crucial for normal biological process through the removal of unrepaired damaged cells [171,172]. Deregulation in apoptotic pathways has been associated with various human diseases, including cancers [173,174,175]. Since the previous decade, our understanding of the miRNA’s role in regulating cell death has increased exponentially. Many reports have linked various apoptotic genes as a direct target of miRNAs and their underlying importance in oral tumor progression and drug resistance. Increased expression of miR-101-3p and miR-199b-5p promoted apoptosis by suppressing BICC1 expression in TSCCA and SSC-9 cells [176]. A study showed that miR-203 induces the apoptosis of YD-38 human oral cancer cells by directly targeting semaphorin 6A (SEMA6A), suggesting its potential application in anticancer therapeutics [122]. Later it was discovered that overexpression of miR-203 significantly increased not only DNA segmentation but also the apoptotic population in YD-38 cells. Microarray analysis revealed that the expression of the polycomb complex protein gene Bmi-1, a representative oncogene, was greatly downregulated by miR-203 in YD-38 cells [121]. Another study reported the functional role of miR-376c-3p in regulating the cell cycle and apoptosis of human oral squamous cancer cells by suppressing homeobox B7 (HOXB7). It was also involved in inducing G1/G0 arrest and directing apoptosis in SCC-25 cells [177].

In another study, miR-26a was found to be overexpressed along with Bim and Bax, in cells treated with metformin. These results suggest that the anti-proliferative nature of metformin in KB human oral cancer cells might result partly due to the induction of apoptosis by downregulation of Mcl-1 levels by miR-26a [67]. It was found that miR-139 can induce apoptosis by regulating the AKT signaling pathway in Tca8113 cells which might lead to the development of a more effective method for the treatment of oral cancer [94]. miR-548d-3p is known to inhibit apoptosis by regulating the JAK signal transducer and activator of transcription (STAT) signaling pathway through binding to the 3′ UTR region of SOCS5 and SOCS6. In a recent study long non-coding RNA maternally expressed gene 3 (MEG3) was shown to promote apoptosis by sponging the levels of miR-548d-3p [178]. Another study showed that overexpression of miR-486-3p led to the growth inhibition and induction of apoptosis which was a similar phenotype observed by knockdown of discoidin domain receptor 1 (DDR1). It led to the conclusion that miR-486-3p functions as a tumor suppressor in oral cancer by targeting DDR1. Moreover, it was also suggested that miR-486-3p has the possibility of being transcriptionally co-regulated with its host gene ANK1 through epigenetic repression when treated with DNA methylation inhibitor elucidating its potent role in targeting apoptosis [150]. miR-214 expression was observed to be elevated and RASSF5 was down-regulated in oral cancer cell lines. Moreover, miR-214 regulated KB cell apoptosis through targeted inhibition of RASSF5 expression, FOXO3a phosphorylation, and BIM expression, suggesting its plausible application as a novel therapeutic oral cancer target [128]. Shang, A. et al. (2018) investigated the functional role of miR-9 in the pathogenesis of oral squamous cell carcinoma. Downregulation of miR-9 was observed in tumor tissues and forced expression of the same promoted apoptosis via targeting cyclin-dependent kinase 4/6 (CDK4/6) proliferative pathways [55]. miR-155 was found to be upregulated in OSCC patients’ samples and further experimentation reported that inhibition of miR-155 directly target p27Kip1, a cell checkpoint inhibitor to induce G1 arrest, increased cleaved caspase-3 activity, and promoted apoptosis in Tca8113 cells [179]. Moreover, other miRNAs (miR-1-3p [54] miR-29a [70], miR-101 [82], miR-205 [125], miR-377 [142], miR-543 [155], miR-4513 [165]) have been found to play a crucial role in regulating apoptosis in oral tumorigenesis. Hence, these results indicate the importance of miRNA in modulating the apoptotic pathways, which result in the progression of oral cancers.

### 3.3. Epithelial-To-Mesenchymal Transition (EMT)

Studies carried out over the past decades have shown the detrimental role of EMT in increasing the morbidity and mortality of human cancers [180,181]. The process, characterized by the loss of cell-cell adhesion, apical–basal polarity, and increment in the motility of cell is known to be controlled by a set of molecules that play the role of effectors, regulators, and inducers of EMT [182,183,184]. A piece of robust transcriptional machinery is essential for monitoring the expression of the epithelial and mesenchymal markers during EMT [185,186]. miRNAs being the regulator of genetic code, plays a pivotal role in the induction of EMT phenotype in oral cancers. miR-155-5p expression might contribute to EMT-associated OSCC progression and serve as a biomarker for predicting relapse, especially for patients with early-stage OSCC. miR-155-5p has a multifaceted role in regulating various EMT machinery by regulating different transcription factors and signaling pathways. A negative correlation was noted between miR-155-5p and E-cadherin expression, suggesting that miR-155-5p plays an important role in EMT. Moreover, miR-155-5p was found to assist EMT either by inducing transforming growth factor β1 or through the phosphoinositide 3-kinase/serum and glucocorticoid-regulated kinase 3/β-catenin signaling pathway. Furthermore, miR-155-5p inhibitor transfected cells showed both, an increase in a suppressor of cytokine signaling 1 (SOCS1) and a decrease in transcription factor signal transducer and activator of transcription 3 (STAT3) in HSC-3 OSCC cells, possibly suggesting that the activation of SOCS1 causing downregulation of STAT3 by inhibiting the action of miR-155-5p. miR-155 also led to the downregulation of BCL6 expression and an increase in cyclin D2 expression. This helped in the proliferation, migration, and invasion of CAL27 OSCC cells as STAT3 generally functions as a tumor promoter in different malignancies [187,188,189,190]. It is already a well-established fact that the BCL6 promotes EMT by the Zinc finger E-box binding homeobox 1 (ZEB1)-mediated transcriptional repression of E-cadherin in breast cancer cells. Thus, the study data from OSCC tissue samples established a quantitative association between miR-155-5p and E-cadherin expression, its relapse, and disease-free survival (DFS). Therefore, highlighting that miR-155-5p is potentially a key modulator to determine the aggressiveness and the chance for relapse in OSCC [104]. Another study established the Yes-associated protein-1 (YAP1) as a direct target gene of miR-27a-3p. An increase in miR-27a-3p could significantly decrease YAP1 expression along with other EMT-associated markers in OSCC cell lines, including Twist and Snail. Further studies revealed that miR-27a-3p downregulated the EMT-related molecules, possibly through the regulation of SRY-box 2 (Sox2) via the YAP1-OCT4-Sox2 signaling axis. This study found that miR-27a-3p could inhibit the YAP1 directly by post-transcription silencing and therefore, potentially suppress the EMT process. Thus miR-27a-3p is an important player for the invasion and metastasis in OSCC through EMT inhibition [68]. Knockdown of miR-29b-1-5p in OSCC suppressed the EMT, which was regained by the forced expression of c-Met. Moreover, cadherin 1 (CDH1) was a direct target of miR-29b-1-5p possibly suggesting that the miR-29b-1-5p acts as an oncogenic miRNA that works in tandem with c-Met to induce EMT in OSCC cells [73]. Another study found that the level of miR-106a decreased significantly, whereas the expression of LIMK1 significantly increased in OSCC cell lines. EMT and proliferation were severely inhibited by the knockdown of LIMK1 in OSCC cells. Luciferase reporter assay confirmed that miR-106a directly targets LIMK1. Thus, the study concluded that there is an inverse relationship between cell proliferation and EMT and LIMK1 expression [84]. Thus, various miRNA’s play a vital role in epithelial to mesenchymal transition in oral cancer leading to tumor progression.

### 3.4. Invasion and Migration 

The dissemination of a tumor cell is a complex phenomenon involving migration and invasion as key characteristic features of metastatic tumors [191,192]. Increasing lines of evidence advocate miRNAs as major players regulating the migration and invasion of oral cancer. Experimental analysis of cancer-associated fibroblast-derived exosomal *miR-382-5p* led to the conclusion that it promoted the invasion and migration of OSCC. Moreover, the cancer-associated fibroblast (CAF) density in tumor tissues was found to be relevant to OSCC lymph node metastasis and the tumor–node–metastasis (TNM) stage. Furthermore, it was revealed that miR-382-5p was overexpressed in CAFs compared with adjacent normal tissue, and upregulation of miR-382-5p was responsible for promoting OSCC cell migration and invasion [144]. miR-196b was significantly overexpressed in OSCC tissues compared with the corresponding adjacent normal tissue samples. Moreover, it was found that the epigenetic regulation of miR-196b expression plays a pivotal role in modulating cell migration and cell invasion during OSCC progression [115]. An analysis of miR-211 led to the conclusion that it promotes invasion and migration ability of OSCC cells via targeting the bridging integrator 1 protein [127]. Another study discovered that miR-29b promoted OSCC cell migration by downregulating CX3CL1, a cell-cell adhesion regulator, which plays a pivotal role in miR-29b-regulated OSCC cell migration machinery [71]. Wei, Z. et al. (2019) demonstrated that the invasion and migration attributes of OSCC cells were drastically reduced after treatment with miR-5100 inhibitor. Upregulation of miR-5100 was observed with concomitant downregulation of suppressor of cancer cell invasion (SCAI) levels in OSCC cells. Moreover, SCAI was verified as a direct target of miR-5100 [166]. 

MiRNAs are also known to inhibit the invasion and migration of oral cancer and could be used as a novel therapeutic strategy for metastatic cancer treatments. Recently, miR-29b-3p was shown to act as a guarder that suppressed the migration of OSCC cells. It was reported that miR-29b-3p regulated IL-32 through AKT signaling via direct binding to the 3′ untranslated region of the IL-32 mRNA transcript [72]. In another study, miR-4513 expression was found to be elevated in the OSCC cell lines and the forced downregulation of miR-4513 expression inhibited cell invasion and migration in OSCC [165]. Similar reports of increased expression of miR-299-3p through introduction of mimics were found to inhibit oral squamous cell carcinoma cells proliferation and migration [135]. Upregulation of miR-543 promoted the invasion and migration of OSCC cell lines suggesting its oncogenic role in oral cancers [155]. In vitro experiments using rescue of miR-377 resulted in repressed cell growth, induced apoptosis, and reduced cell migration. Further analysis revealed miR-377 to directly target the 3′UTR region of HDAC9 mRNA transcripts [142]. Wei, D. et al. (2019) explored the functional role of miR-199a-5p in oral cancer initiation and progression. The findings reported that the overexpression of miR-199a-5p inhibited cell migration and cell invasion, and blocked the epithelial-mesenchymal transition (EMT) cascade [117]. miR-655 is known to suppress invasion along with abrogating proliferation in oral squamous cell carcinoma by targeting metadherin [160]. Lu, L. et al. (2013) explored the role of miR-29a in oral squamous cell carcinoma, and the results showed that exogenous overexpression of miR-29a inhibited OSCC cell invasion and anti-apoptosis significantly by targeting MMP-2. Moreover, knockdown of miR-29a promoted OSCC cell invasion and induced drug-resistance in vitro [70]. MiR-205-5p was found to suppress the invasiveness of oral squamous cell carcinoma by inhibiting tissue inhibitor of metalloproteinase 2 (TIMP2) expression. The results also suggested that TIMP2 promotes tumor progression and miR-205-5p directly regulates TIMP2, which leads to suppression of pro-MMP 2 activations and inhibition of OSCC cell invasiveness [126]. In another study, miR-124 was found to suppress oral squamous cell carcinoma motility by targeting integrin subunit beta 1 (ITGB1) [193]. Some miRNAs (miR-1 [53], miR-10b [57], miR-17/20a [59], miR-22 [64], miR-23b [66], miR-27 [66], miR-31-5p [76], miR-99a-5p [81], miR-146a-5p [100], miR-155-5p [105], miR-188 [111], miR-195-5p [114], miR-200c-3p [119], miR-216a [129], miR-218-5p [131], miR-320 [136], miR-375 [140], miR-450a [147], miR-491-5p [151], miR-495 [153], miR-650 [158]) have been implicated in regulating invasion and migration of oral cancer. Thus, these studies dictate the pivotal role and mechanism of miRNAs in modulating invasion and migration of oral tumor cells in malignancy. 

### 3.5. Metastasis 

Metastasis is the spread of the primary localized tumor to new positions in the body and is the major cause of cancer-related morbidity worldwide [194,195]. Tumors grow and spread by intricate cross-talk between tumor cells, stromal cells, and its extracellular matrix. As miRNAs behave as genetic switches in the physiological process, they are known to be involved in regulating the reprogramming of molecular events associated with metastasis. One of the studies reported that miR-204-5p could enhance OSCC cell proliferation and metastasis. It was predicted as a regulatory miRNA of CXCR4 in OSCC, and the data analysis strongly indicated a negative correlation between miR-204-5p and CXCR4 expression in OSCC tissues from the patients [123]. A study showed that the levels of miR-497 levels were significantly increased and that of SMAD7 were decreased in OSCC patients’ specimens when compared to the adjacent non-tumor tissue. The 5-year survival of the patients with higher miR-497 levels was found to be lower in the surgically resected OSCC samples as compared to healthy controls. In silico analyses showed that miR-497 targeted the 3’-UTR of SMAD7 mRNA to inhibit its translation [154]. Next-generation sequencing for miRNA profiling revealed that miR-21-3p was significantly overexpressed in the OSCC tissues when compared with the corresponding normal tissues. Moreover, high miR-21-3p expression levels were directly correlated with N classification with a p-value of 0.042 [63]. Another study led to the conclusion that miR-99a repressed oral cancer cell migration and invasion partly through decreasing myotubularin related protein 3 (MTMR3) expression. It also led to the discovery that MTMR3 might serve as a therapeutic target for oral cancer treatment [80]. In another analysis, miR-200c was reported to be downregulated in OSCC tissues when compared with adjacent normal tissues. This showed that miR-200c knockdown in the human oral cancer cell line HOC313 significantly suppressed cell invasion and migration, indicating the ability to inhibit tumor progression [118]. In another study, it was analyzed that miR-372 and miR-373 were upregulated in OSCC tissues relative to the control mucosa. Among different clinical variables, overexpression of miR-372 and miR-373 were closely related to nodal metastasis as well as lymphovascular invasion and poor survival. Multivariate analysis showed that both high miR-372 and miR-373 expression were independent predictors for poor survival in OSCC. Further, it was found that miR-372 regulated LATS2 expression in OSCC cell lines [139]. Zhuang et al. (2017) elucidated the functional role of miR-138-5p where it was found to target ΔNp63 which increases growth, metastasis, and stem-like properties of OSCC cells, and depletion of ΔNp63 greatly represses the OSCC cellular phenotypes in vitro as well as in vivo. It was also found that ΔNp63 isoform transcriptionally suppresses the expression of miR-138-5p [93]. In another study, it was found that miR-98 inhibits cancer cell growth and metastasis by direct targeting IGF1R, implicating miR-98 as a novel potential therapeutic target for OSCC [78]. As shown here, various studies have established miRNAs as essential regulators of metastasis by mediating each step of this transformation. 

### 3.6. Chemoresistance

One of the major problems faced by chemotherapeutics for oral cancer is the acquisition of drug resistance causing poor survival outcomes in patients [196,197]. Induction of drug resistance inhibits the apoptosis triggered by the drug leading to increased survival response and proliferation of tumor cells [198]. Recently, miRNAs are associated as essential regulators in the induction of survival responses leading to the acquisition of drug resistance. In one study, exosomes derived from cisplatin-resistant OSCC cells released miR-21 to induce cisplatin resistance in OSCC cells by targeting the phosphatase, tensin homolog, and programmed cell death 4 receptor, as well as led to the decrease in DNA damage signaling response to the cisplatin [199]. The results of another study indicated that STAT3/miR-21 axis could be a plausible therapeutic target for OSCC chemoresistance. It was suggested that STAT3 regulated the OSCC cell survival and confer DNA damage resistance through the upregulation of miR-21 and its subsequent downstream targets, including phosphatase and tensin homolog (PTEN), TIMP3, and PDCD4 [200]. Inhibiting miR-1246 in oral cancer stem cells (OCSC) led to a reduction in the stemness hallmarks of the cells, whereas the overexpression of miR-1246 led to enhancements of these characteristics. Additionally, the downregulation of miR-1246 led to a decrease in chemoresistance in OSCC cell lines. It was verified that miR-1246 inhibited CCNG2 which contributed to the cancer stemness of OSCC [161]. In another study, it was confirmed that miR-654-5p promoted chemoresistance of OSCC in vitro and in vivo. It regulates the OSCC progression, likely, through the GRAP/Ras/Erk signaling pathway suggesting its important role as a potential biomarker for the clinical diagnosis and prognosis of OSCC [159]. Recently, Kirave et al. (2020) established the significance of exosomal-mediated miR-155 shuttling in the cisplatin-induced chemoresistance, commonly observed in OSCC cells. Transfer of extracellular vesicles to the cisplatin sensitive cells from resistant cell lines were found to have a significant increase in miR-155 levels in the recipient sensitive cells. Restoration of miR-155 triggered the epithelial to mesenchymal transition, increased migration potential, and attainment of the resistant phenotype [201]. Thus, these studies indicate the potential of miRNAs in inducing and regulating chemoresistance in oral cancer and their targets could be used to direct the cancer cells to committed stages of cell death.

### 3.7. Radio-Resistance and Radiosensitivity

Various pieces of evidence suggest that oral cancer stem cells (OSCs) possess high tumorigenic and metastatic properties leading to the acquisition of radio-resistance. This in turn leads to disease recurrence and poor clinical outcomes in oral cancer patients. Very few studies have presented miRNAs involvement in radiosensitivity and radio-resistance for oral cancer. Upregulation of miR-494-3p in SAS OSCC cell lines led to an increase in the population of senescence-associated β-galactosidase positive cells with upregulation of p16INK4a and retinoblastoma 1 (RB1) levels and downregulation of Bmi 1 expression. Thus, the study showed that miR-494-3p could increase the radiosensitivity of OSCC cells through the induction of cellular senescence caused by the downregulation of Bmi 1 [152]. A similar mechanistic study showcased that the andrographolide, derived from the medicinal plant Andrographis, increased the expression of miR-218, resulting in the downregulation of Bmi1, hence reducing the stemness of cancer cells. Thus, the results suggest that andrographolide is a viable natural compound for the treatment of OSCC by increasing the radiosensitivity [202]. The downregulated miR-125b expression was associated with proliferation and radio-resistance mechanisms in OSCC, likely through intercellular Adhesion Molecule 2 (ICAM2) signaling. In another study, the expression of miR-125b was confirmed through real-time quantitative reverse transcriptase–PCR and functional analysis revealed that the activity of miR-125b might contribute to suppressing proliferation and overcoming radio-resistance in OSCC [203]. Similarly, in another research, it was demonstrated that miR-375 inhibits growth and enhances radiosensitivity in OSCC cells by targeting IGF-1R. The upregulation of miR-375 caused a significant inhibition in growth, induction of cell cycle arrest in G0/G1 phase, increase in apoptosis, and enhanced radiosensitivity in OSCC cells, overall suggesting that miR-375 may be a potential therapeutic target for OSCC patients [141]. Although few studies potentiate the role of miRNAs in inducing radioresistance in oral cancer, further studies are required to understand the mechanisms and plausible targets to develop a combinatorial therapeutic regimen leading to inhibition of tumor growth. 

## 4. miRNAs as Biomarkers for Oral Cancers 

One of the major causes of morbidity and poor survival of oral cancer patients is the lack of detection at earlier stages [204]. Moreover, the current diagnostic methods, such as oral examination or collection of biopsies, followed by histopathological analysis, pose various drawbacks in terms of tissue heterogenicity, inaccurate stratification, surgical complications leading to painful invasive procedures [205]. Hence, it is imperative to search for non-invasive biomarkers that can detect the tumor at earlier stages and complement the therapeutics to eliminate cancer [206]. miRNAs fit the profile for candidate biomarkers because of their distinct and unique signatures in different cancer types when compared with the adjacent normal [207]. Besides, being in high abundance, most of the miRNAs are secreted in bodily fluids like the serum, saliva, and plasma, which can be convenient for non-invasive diagnostic procedures [208]. Hence the utility of miRNAs as prospective biomarkers should be explored further to establish them as potential biomarkers for the clinical management of this oral malady. 

The use of expression profiling to determine the molecular classification of the type of human cancers has recently gained impetus in discovering novel biomarkers for diagnosis and establishing a therapeutic regimen [209,210]. Several studies have reported the aberrant deregulation of miRNAs in human cancers, including tumor tissues, serum, plasma, and saliva [211,212]. Some of the relevant candidate miRNAs associated with the deregulation in oral cancers have been listed in Table 2. Wong et al. (2008) was the first to detect the aberrant expression of miRNAs in oral cancer. Microarray analysis revealed the overexpression of miR-184 levels in 20 oral tumor tissues as compared to healthy controls. Moreover, miR-184 was found to be upregulated in plasma and its expression was decreased after surgical resection indicating it as a prognostic factor [213]. Further exploration by the other groups potentiated the role of miR-184 in oral cancer, especially at the surgical tumoral margin site advocating its importance in assessing the minimal disease residue [214,215]. Since then, several studies have reported promising miRNAs as biomarkers in different bodily fluids and at the tumor site. A retrospective study indicated the unique signatures of miRNAs from 51 formalin-fixed oral tumor tissues. Upregulation of miR-16, miR-21, miR-423, let-7i, miR-20a, miR-155, miR-106b, and miR-142-3p was observed with concomitant downregulation of miR-375, miR-125b, and miR-10a [216]. Childs et al. (2009) revealed the differential miRNA expressed in oral primary tumor tissues; miR-155, miR-21, miR-221, and miR-191 were found to be overexpressed, whereas let-7d, miR-1, miR-205, and miR-133a were downregulated [217]. Moreover, miR-375 was reported to be under-expressed and miR-200c, miR-21, and miR-34a were overexpressed in tumor tissues when compared to the control tissues [218]. In a multi-cohort study involving 54 oral cancer patients, 7 patients suffering precancerous lesion, and 36 healthy individuals, miR-10b was significantly expressed with receiver operating characteristic (ROC) analysis (area under the curve (AUC) of 0.932 for oral cancer and AUC of 0.967 for precancerous lesions) indicating a high potency for its usage as an oral cancer biomarker [219]. Lamperska et al. (2016) implicated the use of miR-21 and miR-205 to evaluate the clarity in surgical margins of oral tumor tissues, but couldn’t correlate the miRNAs with clinical outcomes in patients [220].

Recently, studies have advocated the exploration of circulating miRNAs present in bodily fluids like the serum, plasma, or saliva, as it offers non-invasive strategies for ameliorating the survival and quality of life of patients making miRNAs as next-generation diagnostic tools. In a study, miR-24 was found to be significantly overexpressed in plasma of oral cancer patients with a typical ROC curve (AUC) of 0.82 with high sensitivity [244]. Similarly, another study showed a positive correlation between overexpressed miR-24 levels in tumor tissues and the clinical stage [245]. High expression of miR-196a and miR-196b was reported in the plasma of OSCC patients. Extensive ROC analysis (AUC of 0.864 and 0.960) suggested these miRNAs be unique and specific to oral cancer patients when compared with the healthy controls [239]. Tachibana et al. (2016) identified 20 aberrant and unique miRNAs from a subset of 1211 human miRNA array in gingival squamous cell carcinoma samples. Interestingly, there were wide variations in the levels of miR-223 in the plasma and tissue of patients. miR-223 exhibited high levels in plasma whereas it was downregulated in tumor tissues which could be due to its release in the bloodstream from the tumor site as a defense mechanism to inhibit cancer growth [133]. A study investigated the deregulated and significant miRNAs in 101 oral cancer patients and 103 healthy individuals. A significant increase in miR-483-5p serum levels was observed which could differentiate cancer patients with high sensitivity and specificity of 85% and 74% respectively. Moreover, its upregulation in serum was positively correlated with the tumor–node–metastasis (TNM) classification and lymph node metastasis suggesting its plausible importance in the stratification of late and early-stage cancer [149]. 

A grade-specific increase in expression of miR-200b-3p has been reported in plasma samples of grade II-III tumors when compared with grade I samples. Moreover, the circulating levels of miR-200b-3p were decreased when the tumor was surgically removed. It exhibited high sensitivity (90%) and specificity (88.75%) in the classification of oral samples from healthy individuals [241]. In another study, miR-187-5p was upregulated in plasma of 63 OSCC patients when compared with 23 healthy samples with an AUC value of 0.73 indicating its high sensitivity [238]. Later, miR-187-5p was associated as an oncogene to increase the proliferation and migration of oral cancer cell lines. A recent study by Lu and his colleagues explored the selected five miRNAs (miR-31-5p miR-99a-5p, miR-21-5p miR-375-3p, and miR-138-5p) based on previous reports and evaluated its expression levels in serum and tissue of oral cancer patients. Increased expression levels of miR-31-5p were observed in both serum and tissue samples, whereas serum levels of miR-375-3p, miR-138-5p, and miR-99a-5p were associated with clinical outcomes [232]. Another study investigated the miRNome of cancer and healthy oral mucosa. miRNA-seq platform analyzed the differentially regulated plasma miRNA in 55 OSCC patients and 18 healthy individuals. Notably, four miRNAs (miR-769-5p, miR-370-3p, miR-144-5p, and miR-30a-5p) showed upregulation in plasma samples from cancer patients. Moreover, the ROC analysis using the combinational approach of two miRNAs, miR-370-3p and miR-30a-5p resulted in an AUC value of one suggesting it as a potential biomarker in oral cancer diagnosis [230]. 

Genome-wide expression analysis of human saliva samples revealed distinct miRNA signatures when compared with healthy controls. Upregulation of miR-24 and miR-27b with decreased expression of miR-125a, miR-125a, miR-1250, miR-668, miR-136, miR-148a, miR-323-5p, miR-147, miR-200a, miR-503, miR-646, miR-877, miR-632, and miR-220a was observed in saliva of OSCC patients [246,247]. Further studies with miR-27b associated its expression with oral lichen planus, a precancerous lesion, and patients with OSCC recurrence [229]. Gai et al. (2018) first reported the profile of salivary miRNAs present in tumor-derived extracellular vesicles (ECVs). The study revealed miR-517b-3p and miR-320-3p as unique miRNAs present in ECVs and increased levels of miR-512-3 and miR-412-3p in oral tissues with AUC values of 0.847 and 0.87, respectively [243]. A phase I observational clinical trials with 360 participants are recruiting to investigate the clinical utility of serum derived miRNAs unique signatures in high-risk oral precancerous lesions (ClinicalTrials.gov Identifier: NCT03202810). Another clinical trial is ongoing to explore the prognostic value of miR-29b in 100 oral cancer patients. Blood and saliva samples will be collected over the period of the study and patient specific information with lifestyle factors will be taken into consideration in this trial (ClinicalTrials.gov Identifier: NCT02009852). In one of the Phase III interventional and randomized clinical trials, 62 participants will be treated with metformin hydrochloride and their miRNA signatures will be evaluated for establishing them as disease monitoring tools (ClinicalTrials.gov Identifier: NCT03685409). These findings suggest miRNAs as plausible candidates to be used in the development of novel diagnostic tools leading to the circumventing of oral tumorigenesis. 

## 5. miRNAs as a Therapeutic Approach for Oral Cancer

Though the recent development of drugs and therapeutic strategies have capacitated improvements in the diagnosis and treatment of oral cancer, the fact that the OSCC is the most common cause of cancer-related death in head and neck cancers worldwide prompts an urgent need for novel and more efficacious therapies [33]. Accumulating evidence on the role of miRNAs in the development and regulation of oral cancers emphasizes the potential of it being used as a therapeutic target [248]. With the advancement of newer forms of technology, such as synthetic biology and nanotechnology, several RNA-guided medicines are being developed and are extensively investigated [26,249]. The most alluring attribute of using miRNA-based therapeutics is its silencing of genes in advanced stages of cancer with utility in cancer detection at any stage. In addition to its specificity, targeting multiple genes in pathways responsible for tumor progression is also an added benefit [250]. The miRNA-based silencing approach has achieved lots of attention in cancer therapeutics due to its fast, economical, and site-specific delivery parameters. The in vitro experimental data shows promising results but optimization needs to be done for onsite delivery of this drug in humans [251]. The main obstacles to achieving gene silencing in vivo arise due to the instability of the RNA molecule, its low transfection efficiency, and distribution in the target tissue [252]. Apart from these limitations, cross-talk with the differential feedback loops, pleiotropic effects of genes, heterogeneity of the tumor, and reoccurrence of the tumor after therapy are other challenges to onsite delivery of these small RNA molecules [253]. A plethora of literature indicated the application of using anti-miRs or miRNA mimics as a novel therapeutic approach, as these molecules can manipulate miRNA expression and function when delivered locally or systemically [254,255]. The identification of crucial miRNAs with oncogenic or tumor-suppressive roles in OSCC has given a newer strategy for their application in OSCC therapy. 

Various in vitro and in vivo investigations have indicated the potential of miRNAs to suppress various tumorigenic hallmarks of oral cancers. In one of the studies, miR-381-3p mimics when transduced into SCC-9 and Tca-8113 cell lines, resulting in the downregulation of FGFR2 leading to inhibition of proliferation and migration [143]. Another study demonstrated that overexpression of miR-205 downregulated ZEB1, ZEB2, and N-cadherin, and upregulated E-cadherin leading to reduced migratory and invasive attributes of oral cancer cells [256]. Overexpression of miR-375 upregulated SCC-4 cell radiation-induced apoptosis by regulation of IGF1R [141]. Fu et al. (2017) demonstrated that downregulation of miR-155 leads to overexpression of CDKN1B, which in turn inhibited cell proliferation and cell cycle progression in oral cancer Tca8113 cells [179]. A study revealed that cells transfected with miR-373-3p mimics exhibited downregulation in E-cadherin and CK18 levels with concomitant upregulation in N-cadherin expression [257]. Jiang et al. (2014) showed that decreased levels of miR-222, induced chemosensitivity of oral cancer cells to cisplatin (CDDP) and suggested a combination of anti-miR-222 and CDDP may result in overexpression of p53, which regulates apoptosis could be a novel targeting approach [258]. Another study found that miR-205 targets the IL-24 promoter directly and induces gene expression. Thus, miR-205 has a great therapeutic potential to turn on silenced tumor suppressor genes [125]. Later, the same group also established that miR-205 suppresses the oral carcinoma oncogenic activity via downregulation of Axin-2 in the KB human oral cancer cell [124]. It was reported that miR-16 functions as a tumor-suppressor gene in oral squamous cell carcinoma by targeting AKT3 and BCL2L2 [58]. Another study observed that miR-125b suppresses oral oncogenicity by targeting the anti-oxidative gene PRXL2A [87]. A study of miR-181a showed its tumor-suppressive effect against oral squamous cell carcinoma cells by downregulating K-ras expression levels [106]. In another study, it was found that miR-338 suppresses the growth and metastasis of OSCC cells by targeting NRP1 [137]. miR-199a 5p was found to function as a tumor suppressor in oral squamous cell carcinoma via targeting the inhibitor of nuclear factor kappa-B kinase subunit beta (IKKβ)/NF-κB signaling pathway [117], which is quite interesting as IKKβ/NF-κB has generally been reported to promote tumorigenesis [259,260,261,262]. An analysis of miR-186 indicated it as a tumor suppressor in oral squamous cell carcinoma by negatively regulating the protein tyrosine phosphatase SHP2 expression [109]. Though a number of Phase I clinical trials with systemically administered miRNA molecules conjugated with different delivery vehicles are already complete for other chronic diseases, further clinical studies are imperative to establish these new therapeutic efficacies in successfully and safely inhibiting targeted gene products in patients with oral cancer. 

## 6. Conclusions

It is now evident that miRNAs play a pivotal role in regulating different hallmarks of oral tumorigenesis, such as proliferation, apoptosis, invasion, migration, and metastasis. Moreover, recent research associate miRNA aberrant signatures in modulating chemoresistance and radioresistance in oral cancer. miRNA regulatory network should be considered as an intricate cross talk between the target mRNA and miRNA leading to post transcriptional inactivation. The emergence of new miRNA knowledge and its potential role in the cancer creates a new understanding of cell transformation. Hence, it is necessary to further study about the different roles of miRNAs, which can contribute to early diagnosis, targeted therapy, and prognosis evaluation of oral cancer patients. Though cancer-related miRNome repertoire is ever-increasing, there exists a large-scale variation among the results of different studies. These variations could be due to change in study design, population size, use of relevant controls (either adjacent normal or healthy control), methodology, pool size, tumor heterogenicity, variability in ethio–physiology. Careful and logical selection, along with functional characterization of miRNAs, is very crucial for understanding the dynamics of miRNA regulation. Standardized and randomized validation research must be undertaken to ensure the sensitivity, specificity, and robustness of the miRNA studies for tailoring individual patient conditions leading to development of personalized therapeutic regimen. Thus, it is necessary to do well-designed, multi-centered trials with large patient groups, to mitigate external variations in data sets. This will provide useful and accurate information for development of novel diagnostics and pave the way for more detailed and precise studies on miRNAs and OSCC in general. Identifying basal threshold levels or combining differentially expressed miRNAs could pave the way for development of early diagnostic and prognostic tools. Many possibilities present for miRNA in oral cancers include targeting genes that appear to be mediators in cancer progression, discovering novel biomarkers for early diagnosis, identifying molecular targets, and engineering delivery vehicles conjugated with DNA as therapeutic devices; thus, representing the ideal theranostic approach. A better understanding of the putative miRNA targets through *in silico* pathway and validation analysis would open up different perspectives for more refined and effective therapeutic regimens combating oral cancer.

## Figures and Tables

**Table 1:** Different microRNAs and their targets involved in oral cancer initiation and progression.

miRNA	Target Gene	Mechanism or Functions	Reference
miR-1 ↓	SLUG	↑ Tumorigenicity and invasiveness	[53]
miR-1-3p	DKK1	↓ Transit of SCC-4 cells from G0/G1 to S and ↑ apoptosis	[54]
miR-9 ↓	CDK4/6 ↓	↑ Cell-cycle arrest at G0/G1 and ↑ apoptosis	[55]
miR-10a↑	GLUT1	↑ Cell proliferation and glucose metabolism	[56]
miR-10b ↑		↑ Cell migration and invasion	[57]
miR-16 ↓	AKT3 and BCL2L2	↓ Tumor	[58]
miR-17/20a ↓	ITGβ8	↓ Cell migration	[59]
miR-18a-5p↑	Smad2	↓ E-cadherin, and ↑ Smad7 TGFβ, αSMA, vimentin.	[60]
miR-21	TNF-α	Cell proliferation	[61]
miR-21 ↑		↑ Tumor size, metastasis and local invasion	[62]
miR-21-3p ↑		↑ Metastasis	[63]
miR-22 ↓	NLRP3	↓ Cell proliferation, migration	[64]
miR-23a-3p ↓	FGF2 ↑	↓ Cell proliferation	[65]
miR-23b ↓	MET ↑	↓ Cell migration, invasion	[66]
miR-26a	MCL-1 ↓	↑ Apoptosis	[67]
miR-27a-3p	YAP1	↓ EMT	[68]
miR-27b ↓	MET ↑	↓ Cell migration, invasion	[66]
miR-27b ↓	TCTP ↓	Novel plasma biomarker	[69]
miR-29a↓	MMP2 ↑	↓ Cancer invasion and anti-apoptosis	[70]
miR-29b	CX3CL1 ↓	↑ Cell migration and tumorigenesis	[71]
miR-29b-3p ↓	IL32/AKT	↓ Migration and invasion of OSCC cells	[72]
miR-29b-1-5p ↑	CDH1	↑ EMT	[73]
miR-31 ↑	CXCL12 ↓	↑ Progression from pre-cancer to cancer	[74]
miR-31 ↑	SIRT3	↓ Mitochondrial activity and ↑ oxidative stress	[75]
miR-31-5p	ACOX1	↑ Tumorigenic fitness and ↑cell migration and invasion	[76]
miR-34a ↓	IL6R	↓ Cell proliferation, G1 phase arrest, metastasis and EMT	[77]
miR-98 ↓	IGF1R	↓ Tumor cell growth and metastasis	[78]
miR-99a ↓	mTOR	↑ Growth and survival of OSCC	[79]
miR-99a ↓	MTMR3 ↑	↑ Anti-metastasis	[80]
miR-99a-5p↓	NOX4↑	↓ Proliferation, migration, and invasion	[81]
miR-101↓	ZEB1	↓ Growth, metastasis, and apoptosis resistance	[82]
miR-101-3p ↓	COL10A1 ↑	↓ Proliferation, invasion, and migration	[83]
miR-106a↓	LIMK1	↓ Proliferation and EMT	[84]
miR-107 ↓	TRIAP1	↓ Cell proliferation and migration	[85]
miR-124 ↓	CCL2 and IL-8	↓ Tumor growth	[86]
miR-125b ↓	PRXL2A ↑	↓ Oral oncogenicity	[87]
miR-126 ↓	VEGF-A	↑ Angiogenesis and lymphangiogenesis	[88]
miR-133a-3p ↓	COL1A1 ↑	↓ Proliferation, invasion, and mitosis	[89]
miR-134 ↑	PDCD7 ↓	↓ E-cadherin expression	[90]
miR-138 ↓	YAP1	↓ Tumor and proliferation	[91,92]
miR-138-5p ↓	ΔNp63 ↑	↓ Growth, metastasis, and cancer stemness	[93]
miR-139		↑ Apoptosis through AKTsignaling	[94]
miR-139-5p ↓	HOXA9 ↑	↓ Tumorigenesis and progression	[95]
miR-143 ↓	HK2	↓ Growth of OSCC	[96]
miR-145 ↓	c-Myc and CDK6	↓ Cell proliferation and ↑ G1 phase arrest	[97]
miR-145-5p ↓		↑ Effects of photodynamic therapy and phototoxicity	[98]
miR-146a ↓	SOX2	↓ Aggressiveness of OSCC	[99]
miR-146a-5p ↑	TRAF6 ↓	↑ Proliferation, ↓ TGF-β signaling	[100]
miR-148b-3p	RALBP1	↓ Tumor	[101]
miR-155 ↑		↓ Prognosis	[102]
miR-155 ↑	CDC73 ↓	↑ Cell proliferation	[103]
miR-155-5p		↓ E-cadherin	[104]
miR-155-5p ↑	ARID2	↑ Proliferation, migration, and invasion	[105]
miR-181a ↓	K-ras	↓ Tumor, K-ras protein level, and luciferase activity of vectors	[106]
miR-182-5p ↑	CAMK2N1	↑ Growth, ↓ activation of AKT, ERK1/2, and NF-κB	[107]
miR-184	UCA1 and SF1	↓ Cell proliferation	[108]
miR-186 ↓	PTPN11	↓ Tumor, ↓ signaling of ERK, and AKT	[109]
miR-186 ↓		Potential biomarker	[110]
miR-188 ↓	SIX1	↓ Proliferation and invasion	[111]
miR-194 ↓	AGK	↓ Cell proliferation and inhibits PI3K/AKT/FoxO3a signaling pathway	[112]
miR-195-3p	CCL4	↓ VEGF-C expression and lymphangiogenesis	[113]
miR-195-5p ↓	TRIM14	↓ Proliferation, migration, and invasion	[114]
miR-196b ↑		↑ Migration and Invasion	[115]
miR-199a-5p ↓	SOX4	↓ Migration and invasion of cells via targeting SOX4	[116]
miR-199a-5p ↓	IKKβ	↓ Tumor via IKKβ/NF-κB signaling pathway, ↑ G0/G1 cell cycle arrest	[117]
miR-200c ↓	HOC313	↓ Tumor metastasis	[118]
miR-200c-3p	CHD9 and WRN	↑ Invasion of OSCC	[119]
miR-203 ↓	YES-1 ↑	↓ Oncogenic activity and ↑ apoptosis	[120]
miR-203 ↓	BMI-1	↑ Apoptosis	[121]
miR-203	SEMA6A	↑ Apoptosis	[122]
miR-204-5p ↓	CXCR4 ↑	↓ Proliferation and metastasis of OSCC cells	[123]
miR-205 ↓	AXIN2 ↑	↓ Oral carcinoma oncogenic activity	[124]
miR-205 ↓	IL-24	↑ Apoptosis	[125]
miR-205-5p ↓	TIMP2	↓ Invasiveness, regulates TIMP2 gene and activates proMMP2	[126]
miR-211 ↑	BIN1	↑ Proliferation, migration, and invasion, can inhibit the EGFR/MAPK pathway	[127]
miR-214 ↑	RASSF5 ↓	↓ FOXO3a phosphorylation, BIM expression, caspase 3, and apoptosis	[128]
miR-216a	EIF4B	↓ Proliferation, migration, and invasion	[129]
miR-218 ↑	PPP2R5A	↑ Cisplatin resistance via the PPP2R5A/Wnt signaling pathway	[130]
miR-218-5p ↓	CD44-ROCK	↑ Invasion by targeting the CD44-ROCK pathway	[131]
miR-221 ↓	TIMP3 ↑	↑ Sensitivity of OSCC to Adriamycin	[132]
miR-223 ↑		Novel diagnostic biomarker	[133]
miR-223 ↑	FBXW7 ↓	↑ Proliferation	[134]
miR-299-3p↓	FOXP4	↓ Proliferation and migration, ↑ apoptosis	[135]
miR-320 ↓	NRP1	↓ Migration, adhesion, and tube formation of vascular endothelial cells	[136]
miR-338 ↓	NRP1	↓ Growth and metastasis	[137]
miR-340 ↓	GLUT1 ↑	↑ Lactate secretion, glucose uptake rate, and proliferation of OSCC	[138]
miR-372 ↑	LATS2	↓ LATS2 expression	[139]
miR-373 ↑	LATS2	↓ Survival rate	[139]
miR-375 ↓	PDGFA	↓ Cell Migration and invasion by targeting platelet-derived growth factor A	[140]
miR-375 ↓	IGF-1R	↓ Growth and enhances radiosensitivity, ↑ cell cycle arrest in G0/G1 phase	[141]
miR-377 ↓	HDAC9	↓ Growth, migration, and apoptosis	[142]
miR-381-3p	FGFR2 ↓	↓ Proliferation and cell cycle progression	[143]
miR-382-5p		↑ Migration and invasion	[144]
miR-424-5p ↑	SOCS2 ↓	↑ Oncogenic activity by ↓ SOCS2	[145]
miR-429 ↓	ZEB1	↓ Growth of OSCC	[146]
miR-450a ↑	TMEM182	↑ Motility, ↓ cell adhesion ability, and ↑ invasiveness	[147]
miR-455-5p ↑	UBE2B ↓	↑ Proliferation and tumorigenesis	[148]
miR-483-5p		Novel diagnostic biomarker	[149]
miR-486-3p ↓	DDR1 ↑	↓ Tumor	[150]
miR-491-5p ↓	GIT1 ↑	↓ Migration, invasion, and lung metastasis	[151]
miR-494 ↑		Potential biomarker	[110]
miR-494-3p	Bmi1 ↓	↑ Cellular senescence and ↑ radiosensitivity	[152]
miR-495 ↓	Notch1	↓ Cell proliferation and invasion	[153]
miR-497	SMAD7	↑ Metastasis	[154]
miR-543 ↑	CYP3A5	↑ Proliferation, invasion, and migration, ↓ apoptosis	[155]
miR-545 ↓	RIG-I	↓ Tumor	[156]
miR-596	LGALS3BP	↓ Tumor	[157]
miR-650 ↑	GFI1	↑ Proliferation, migration, and invasion	[158]
miR-654-5p ↑	GRAP ↓	↑ Metastasis and chemoresistance, activates Ras/MAPK signaling and EMT	[159]
miR-655-3p ↓	MTDH	↓ Cell proliferation and invasion by inhibiting PTEN/AKT signaling	[160]
miR-1246 ↑	CCNG2	↑ Cancer stemness and chemoresistance	[161]
miR-1246	DENND2D	↑ Cell motility	[162]
miR-1246 ↑		↓ Prognosis of OSCC	[163]
miR-1254 ↓	CD36	↓ Tumor	[164]
miR-3651 ↑		Potential biomarker	[110]
miR-4513 ↑	CXCL17	↑ Cell proliferation, migration, and invasion, promotes apoptosis	[165]
miR-5100 ↑	SCAI ↓	↑ Proliferation, migration, and invasion	[166]

The arrows illustrated in the first column of the table represent the expression levels of miRNA in oral cancer tissues/cell lines. The second column represent the targets of miRNA found in various studies. The third column indicates the mechanism of action of these miRNAs in modulating various hallmarks of oral tumorigenesis. ↑ denotes increase and ↓ depicts decrease in either in expression/ function of miRNAs.

**Table 2:** MicroRNAs as diagnostic biomarkers in oral cancer.

miRNA	Source	Expression in OSCC	References
Let-7b	Serum	High	[221]
Let-7d	Serum	Low	[221]
miR-7	Serum	High	[221]
miR-9	Serum	Low	[222,223]
miR-16	Serum	High	[221]
miR-16	Tissue	High	[216]
miR-20a	Tissue	High	[216]
miR-21	Blood	High	[224]
miR-21-3p	Tissue	High	[225]
miR-24	Blood	High	[226]
miR-24-3p	Saliva/ECVs	High	[227]
miR-25	Serum	High	[221]
miR-26a	Plasma	High	[228]
miR-27b	Saliva	High	[229]
miR-29a	Serum	Low	[221]
miR-29a	Blood	Low	[226]
miR-30a-5p	Plasma	High	[230]
miR-31	Saliva	High	[231]
miR-31-5p	Serum	High	[232]
miR-96-5p	Tissue	High	[225]
miR-99b	Plasma	High	[233]
miR-125b	Tissue	Low	[216]
miR-125b	Tissue	Low	[203]
miR-130-3p	Tissue	High	[225]
miR-141-3p	Tissue	High	[225]
miR-142	Serum	Low	[221]
miR-142-3p	Tissue	High	[216]
miR-144-5p	Plasma	High	[230]
miR-146a	Tissue	High	[234]
miR-150-5p	Plasma	Low	[221]
miR-155	Blood	High	[235]
miR-155	Tissue	High	[102]
miR-181	Tissue	High	[236]
miR-184	Tissue	High	[213]
miR-184	Serum	High	[237]
miR-187-5p	Plasma	High	[238]
miR-191	Blood	High	[235]
miR-192-5p	Plasma	Low	[233]
miR-194-5p	Plasma	High	[233]
miR-195	Serum	High	[221]
miR-196a	Plasma	High	[239]
miR-196b	Plasma	High	[239]
miR-196a/b	Tissue	High	[240]
miR-200b-3p	Plasma	High	[241]
miR-205	Tissue	Low	[217]
miR-211	Tissue	Low	[242]
miR-212-3p	Plasma	High	[233]
miR-214-3p	Plasma	High	[233]
miR-223	Tissue	Low	[133]
miR-335-5p	Plasma	High	[233]
miR-338	Serum	Low	[221]
miR-370-3p	Plasma	High	[230]
miR-375	Tissue	Low	[216]
miR-375	Plasma	Low	[228]
miR-412-3p	Saliva/ECVs	High	[243]
miR-483-5p	Serum	High	[149]
miR-486-5p	Plasma	Low	[228]
miR-491-5p	Tissue	Low	[151]
miR-494	Blood	High	[235]
miR-512-3p	Saliva/ECVs	High	[243]
miR-601	Plasma	Low	[233]
miR-603	Plasma	High	[233]
miR-624	Serum	High	[221]
miR-660-5p	Plasma	High	[233]
miR-769-5p	Plasma	High	[230]
miR-1303	Plasma	High	[233]
miR-3651	Blood	High	[110]

## Abbreviations

AGO	Argonaute;
AKT	Protein kinase B;
AUC	Area Under Curve;
BCL2L2	Bcl-2-like protein 2;
BIN1	Bridging integrator 1 Protein;
BMI-1	B lymphoma Mo-MLV insertion region 1 homolog;
CAF	Cancer-associated fibroblast;
CCNG	Cyclin G;
CDDP	Cisplatin;
CDH1	Cadherin 1;
CDKN	Cyclin-dependent protein kinases;
COL10A1	Collagen Type X Alpha 1 Chain;
COL1A1	Collagen type 1 alpha 1;
CX3CL1	Chemokine ligand 1;
CXCL17	Chemokine (C-C motif) ligand 17 (CCL17);
CYP3A5	Cytochrome P450 family 3 subfamily A member 5;
DDR1	Discoidin Domain Receptor 1;
DFS	Disease-free survival;
DGCR8	DiGeorge syndrome chromosomal (or critical) region 8;
DNA	Deoxyribonucleic Acid;
EMT	Epithelial-to-Mesenchymal Transition;
FAP	Familial adenomatous polyposis;
FGF2	Fibroblast growth factor 2;
FGFR	Fibroblast growth factor;
FGFR2	Fibroblast growth factor receptor 2;
FOXP4	Forkhead box P4;
GLUT1	Glucose Transporter 1;
GRAP	GRB2 related adaptor protein;
GTP	Guanosine triphosphate;
HDAC	Histone deacetylase;
HNSCC	Head and neck squamous carcinoma;
HOXA10	Homeobox A10;
HOXB7	Homeobox B7;
HPV	Human papillomavirus;
ICAM	Intercellular adhesion molecule;
IGF	Insulin-like growth factor;
IL32	Interleukin 32;
ITGB1	Integrin subunit beta 1;
LATS2	Large tumor suppressor, homolog 2;
LIMK1	LIM Domain Kinase 1;
MEG3	Maternally expressed gene 3;
miRISC	miRNA induced silencing complex;
miRNAs	MicroRNAs;
MMP2	Matrix metalloproteinase-2;
mRNA	Messenger RNA;
MTMR3	Myotubularin related protein 3;
OSCC	Oral Squamous Cell Carcinoma;
PCNA	Proliferating cell nuclear antigen;
PDCD	Pyruvate dehydrogenase complex deficiency;
PTEN	Phosphatase and tensin homolog;
RASSF5	Ras Association Domain Family Member 5;
RB1	Retinoblastoma susceptibility gene;
RISC	RNA-induced silencing complex;
RNA	Ribonucleic acid;
ROC	Receiver operating characteristic;
SCAI	Suppressor of cancer cell invasion;
SCC	Squamous cell carcinoma;
SEMA6A	Semaphorin 6A;
SHP2	SH2 containing protein tyrosine phosphatase-2;
SOCS1	Suppressor of cytokine signaling 1;
STAT	Signal transducer and activator of transcription;
TIMP	Tissue inhibitor of metalloproteinase;
TME	Tumor microenvironment;
TNF-α	Tumor Necrosis Factor-α;
TRIAP1	TP53 regulated inhibitor of apoptosis 1;
TS-miRs	Tumor suppressor micro RNAs;
UCA1	Urothelial cancer-associated 1;
UTR	Untranslated regions;
YAP1	Yes-associated protein-1;
ZEB1	Zinc finger E-box binding homeobox;
SCAI	Suppressor of cancer cell invasion;
FGFR2	Fibroblast growth factor receptor 2;
FOXP4	Forkhead Box P4;
TRIAP1	TP53 Regulated Inhibitor of Apoptosis 1;
SIRT3	Sirtuin (silent mating type information regulation 2 homolog) 3;
IL32/AKT	AKT serine/threonine kinase 1;
CXCL17	Chemokine (C-C motif) ligand 17 (CCL17);
CDH1	Cadherin-1;
ARID2	AT-rich interactive domain-containing protein 2;
CD36	Cluster of differentiation 36;
CCNG2	Cyclin G2;
CYP3A5	Cytochrome P450 Family 3 Subfamily A Member 5;
SMAD7	Mothers against decapentaplegic homolog 7;
c-Myc	Cellular proto-oncogene;
ZEB1	Zinc Finger E-Box Binding Homeobox 1;
BIN1	Bridging Integrator-1;
SFRP1	Secreted frizzled-related protein 1;
IL6R	Interleukin 6 receptor;
HOXA10	Homeobox protein Hox-A10;
HDAC9	Histone Deacetylase 9;
MTDH	Metadherin;
CDK4/6	Cyclin-dependent kinase 4/6;
COL10A1	Collagen Type X Alpha 1 Chain;
TCTP	Translationally-controlled tumor protein;
YAP1	Yes-associated protein 1;
IKKβ	Inhibitor of nuclear factor kappa-B kinase subunit beta;
MMP2	Matrix Metallopeptidase 2;
MET	Proto-Oncogene, Receptor Tyrosine Kinase
SOX4	SRY-Box Transcription Factor 4;
LIMK1	LIM Domain Kinase 1;
TRIM14	Tripartite motif-containing protein 14;
GRAP	GRB2 Related Adaptor Protein;
TIMP3	Tissue inhibitor of metalloproteinase 3;
COL17	Collagen Type XVII Alpha 1 Chain;
SIX1	Sine oculis homeobox homolog 1;
VEGF-A	Vascular endothelial growth factor A;
STAT3	Signal transducer and activator of transcription 3;
EIF4B	Eukaryotic Translation Initiation Factor 4B;
RASSF5	Ras Association Domain Family Member 5;
CD44	Cluster of differentiation 44;
BMI-1	B lymphoma Mo-MLV insertion region 1 homolog;
CHD9	Chromodomain Helicase DNA Binding Protein 9;
WRN	Werner syndrome RecQ like helicase;
PPP2R5A	Protein phosphatase 2 regulatory subunit B alpha;
CXCL12	C-X-C Motif Chemokine Ligand 12;
AXIN2	Axis inhibition protein 2;
UBE2B	Ubiquitin-conjugating enzyme E2 B;
GFI1	Growth Factor Independent 1;
MTMR3	Myotubularin-related protein 3;
TRAF6	TNF Receptor Associated Factor 6;
SOCS3	Suppressor of Cytokine Signaling 3;
STAT3	Signal Transducer and activator of transcription 3;
DDR1	Discoidin Domain Receptor 1;
PDCD7	Programmed Cell Death 7;
HK2	Hexokinase 2;
TMEM182	Transmembrane Protein 182;
SEMA6A	Semaphorin 6A;
RhoA	Ras Homolog A;
IL24	Interleukin 24;
CCL4	C-C Motif Chemokine Ligand 4;
TIMP2	Tissue inhibitor of metalloproteinase 2;
TRIM14	Tripartite Motif Containing Protein 14;
WNT10B	Wingless-type MMTV integration site family, member 10B;
YES1	YES Proto-Oncogene 1;
CD44-ROCK	Rho Assisted Protein Kinase;
CAMK2N1	Calcium/Calmodulin Dependent Protein Kinase 2 Inhibitor 1;
OSM	Oncostatin M;
RALBP1	RalA binding protein 1;
COL1A1	collagen type 1 alpha 1;
LATS2	Large tumor suppressor kinase 2;
MCL1	Induced Myeloid leukemia cell differentiation protein;
TCF12	Transcription Factor 12;
NLRP3	NLR Family Pyrin Domain Containing 3;
AKT3	AKT Serine/Threonine Kinase 3;
BCL2L2	BCL2 Like protein 2;
IGF-1R	Insulin Like Growth Factor 1 Receptor;
CXCR4	C-X-C Chemokine receptor 4;
HOXB7	Homeobox B7;
PTPN11	Tyrosine-Protein phosphatase non-receptor type 11;
DKK1	Dickkopf WNT signaling pathway inhibitor 1;
LGALS3BP	Galectin 3 Binding Protein;
YAP1	Yes Associated Protein 1;
GIT1	G protein-coupled receptor kinase-interacting protein 1;
DENND2D	DENN Domain Containing 2D;
FBXW7	F-box/WD repeat-containing protein 7;
NOX4	NADPH Oxidase 4;
PRXL2A	Peroxiredoxin Like 2A;
SOX2	SRY-box 2;
NOTCH1	Notch homolog 1;
mTOR	Mechanistic target of rapamycin;
HOXA9	Homeobox A9;
NRP1	Neuropilin 1;
ITGβ8	Integrin β8;
SMAD2	Mothers against decapentaplegic homolog 2;
PDGF-A	Platelet derived Growth Factor Subunit A;
AGK	Acylglycerol Kinase;
SOCS2	Suppressor of Cytokine Signaling 2;
BARX2	Homeobox protein BarH-like 2;
CDC73	Cell Division Cycle 73;
ZEB1	Zinc Finger E-Box Binding Homeobox 1;
K-ras	Kirsten rat sarcoma viral oncogene homolog;
NLK	Nemo Like Kinase;
EZH2	Enhancer of Zeste Homolog 2;
PLD1	Phospholipase D1;
ICAM2	Intercellular Adhesion Molecule 2;
MCPH1	Microcephalin;
PUMA	p53 upregulated modulator of apoptosis;
SNAI2	Snail Family Transcriptional Repressor 2 (Slug)
